# Longitudinal Variations of CDC42 in Patients With Acute Ischemic Stroke During 3-Year Period: Correlation With CD4^+^ T Cells, Disease Severity, and Prognosis

**DOI:** 10.3389/fneur.2022.848933

**Published:** 2022-04-25

**Authors:** Xiao Cheng, Jianxin Ye, Xiaolei Zhang, Kun Meng

**Affiliations:** ^1^Department of Neurology, ShanXi Province People's Hospital of Shanxi Medical University, Taiyuan, China; ^2^Shanxi Key Laboratory of Brain Disease Control, Shanxi Provincial People's Hospital, Taiyuan, China; ^3^Department of Neurology, The 900th Hospital of the Joint Logistics Support Force of the Chinese People's Liberation Army, Fuzhou, China

**Keywords:** cell division cycle 42, acute ischemic stroke, NIHSS, Th1/2/17 cells, prognosis

## Abstract

**Objective:**

Cell division cycle 42 (CDC42) modulates CD4^+^ T-cell differentiation, blood lipids, and neuronal apoptosis and is involved in the pathogenesis of acute ischemic stroke (AIS); however, the clinical role of CDC42 in AIS remains unanswered. This study aimed to evaluate the expression of CDC42 in a 3-year follow-up and its correlation with disease severity, T helper (Th)1/2/17 cells, and the prognosis in patients with AIS.

**Methods:**

Blood CDC42 was detected in 143 patients with AIS at multiple time points during the 3-year follow-up period and in 70 controls at admission by reverse transcription-quantitative polymerase chain reaction (RT-qPCR). In addition, blood Th1, Th2, and Th17 cells and their secreted cytokines (interferon-γ (IFN-γ), interleukin-4 (IL-4), and interleukin-17A (IL-17A)) in patients with AIS were detected by flow cytometry and enzyme-linked immunosorbent assay (ELISA), respectively.

**Results:**

Compared with controls (*p* < 0.001), CDC42 was reduced in patients with AIS. CDC42 was negatively correlated with the National Institutes of Health Stroke Scale (NIHSS) score (*p* < 0.001), whereas, in patients with AIS (all *p* < 0.050), it was positively associated with Th2 cells and IL-4 but negatively correlated with Th17 cells and IL-17A. CDC42 was decreased from admission to 3 days and gradually increased from 3 days to 3 years in patients with AIS (*P*<0.001). In a 3-year follow-up, 24 patients with AIS recurred and 8 patients died. On the 3rd day, 7th day, 1st month, 3rd month, 6th month, 1st year, 2nd year, and 3rd year, CDC42 was decreased in recurrent patients than that in non-recurrent patients (all *p* < 0.050). CDC42 at 7 days (*p* = 0.033) and 3 months (*p* = 0.023) was declined in reported deceased patients than in survived patients.

**Conclusion:**

CDC42 is used as a biomarker to constantly monitor disease progression and recurrence risk of patients with AIS.

## Introduction

Stroke is a common cerebrovascular disease, which has affected nearly 104 million people worldwide in the past three decades and has climbed to the second leading cause of death (second only to ischemic heart disease), besides, stroke is also known for its high disability rate (nearly 33.4–71%) (1–4). Acute ischemic stroke (AIS) is the primary type of stroke (accounting for ~70% of all stroke cases), which is characterized by immune system disorder, severe neurological deficits, etc. (5–10). Gradually, diversified therapeutic strategies (including thrombolysis, antiplatelet treatment, anticoagulants, and neuroprotective agents) have been introduced in an attempt to eliminate arterial occlusion, restore blood flow to the brain, and improve the recovery of neurological function; however, AIS is disease prone to recurrence, which requires continuous attention (11–16). Therefore, it is imperative to develop objective biomarkers to help identify patients with AIS as soon as possible, predict their outcomes, and then adjust the treatment regimens accordingly.

Cell division cycle 42 (CDC42), a small hydrolase of guanosine triphosphate (GTPase), acts as a signal transduction convergence point that mediates many signaling pathways. Moreover, it is reported that CDC42 regulates blood lipids, blood vessel development, CD4^+^ T-cell differentiation, microglial process, and neuronal apoptosis in some cardiovascular and cerebrovascular diseases (including ischemic brain injury, coronary heart disease, and cerebrovascular malformations) (17–23). For instance, a study found that CDC42 can act as an upstream activator of the c-Jun N-terminal kinase (JNK) signaling pathway to govern neuronal apoptosis in ischemic brain injury (19). Another study explored the correlation between CDC42 and T helper (Th) 2 cells, Th17 cells, and blood lipids in patients with coronary heart disease (20). Interestingly, these CDC42-modulated biological processes (mentioned above) behave as underlying pathogenesis of AIS, implying that CDC42 might be implicated in the development of AIS (6, 8, 24). Additionally, an *in vitro* study reported that the activation of CDC42 promoted the migration of endogenous neural stem/progenitors cells after ischemic stroke, which facilitates the recovery of injured brain tissue (25). However, the detailed clinical role of CDC42 in patients with AIS remains unanswered.

In this study, the expression of CDC42 was detected in patients with AIS during a 3-year follow-up period with the aim of evaluating the longitudinal changes of CDC42 and its correlation with disease severity, Th1/2/17 cells, and the prognosis in patients with AIS.

## Methods

### Subjects

From September 2016 to March 2018, a total of 143 patients with first ever stroke was consecutively reflected in this study. Included patients met the following criteria: (1) a new diagnosis of AIS confirmed by cranial CT scan or magnetic resonance angiography; (2) age over 18 years old; and (3) receiving treatment in our hospital within 24 h after the stroke episode. Patients who (1) had intracranial hemorrhage, (2) were accompanied by autoimmune diseases or inflammatory disease, (3) were complicated with hematological diseases or malignancies, (4) had a history of stroke, and (5) were lactating women or pregnant women, were excluded. Furthermore, this study also recruited 70 subjects with at least two high-stroke-risk factors as controls [the high-stroke-risk factors were defined in a previous study (26), specifically including hypertension, atrial fibrillation, diabetes mellitus, dyslipidemia, smoking, lack of exercise, overweight, self-reported family, and a history of transient ischemic attack]. All controls were required to have no history of stroke, and the exclusion criteria of AIS were also appropriate for controls. The ethical permission was acquired from the Ethic Committee with Approval Number 72 (2021) from Province People's Hospital. A written informed consent form was provided by each subject or by the corresponding guardian.

### Data Collection and Sample Collection

After inclusion, the clinical data of patients were explained. The National Institutes of Health Stroke Scale (NIHSS) was applied to assess the severity of the disease, which was classified into normal (0–1 point), mild stroke (2–4 points), moderate stroke (5–15 points), moderate-to-severe (16–20 points) stroke, and severe stroke (21–42 points). Trial of ORG 10172 in Acute Stroke Treatment (TOAST) classification was implemented to identify the stroke subtype (27). All patients received usual treatments, including intravenous thrombolysis (IVT) and mechanical thrombectomy. Peripheral blood (PB) samples were collected from each patient immediately after admission. Subsequently, serum samples were separated by centrifugation, and PB mononuclear cells (PBMCs) were separated by the Ficoll density gradient centrifugation. The blood samples of controls were also collected after enrollment, and the PBMC was also divided. Furthermore, for patients with AIS, additional PB samples were collected at the following time points: 1, 3, and 7 days after admission, and 1 month, 3 months, 6 months, 1 year, 2 years, and 3 years at the follow-up. As described above, PBMC was also isolated. In addition, the modified Rankin Scale (mRS) score of patients at 3 months was assessed and collected for study analysis.

### Determination of CDC42

The expression of cell division cycle 42 in PBMCs of patients with AIS and controls was established by RT-qPCR. In brief, PureZOL RNA isolation reagent (Bio-Rad, Hercules, CA, USA) was used to extract the total RNA. Then, iScript™ Reverse Transcription Supermix (Bio-Rad, Hercules, CA, USA) was used to complete the reserve transcription. Subsequently, the qPCR reaction was completed utilizing the QuantiNova SYBR Green PCR kit (QiaGen, Duesseldorf, Nordrhein-Westfalen, Germany). The expression of CDC42 was measured by the 2^−ΔΔCt^ method using glyceraldehyde-3-phosphate dehydrogenase (GAPDH) as an internal reference. Primer sequences were listed in a previous study (20).

### Determination of Cytokines and Th Cells

For patients with AIS, serum cytokines, including interferon- γ (IFN-γ, secreted by Th1 cells), interleukin-4 (IL-4, secreted by Th2 cells), and interleukin-17A (IL-17A, secreted by Th17 cells), were determined by using enzyme-linked immunosorbent assay (ELISA) kits: IFN-γ Human ELISA kit (Invitrogen, Carlsbad, CA, USA), IL-4 Human ELISA kit (Invitrogen, Carlsbad, CA, USA), and IL-17A Human ELISA kit (Invitrogen, Carlsbad, CA, USA). In addition, fresh PBMC samples of 78 patients isolated at admission were used to determine the proportion of Th cells (Th1/2/17) in CD4^+^ T cells by a flow cytometric analysis using the Human Th1/Th2/Th17 Phenotyping kit (BD, Franklin Lakes, NJ, USA). The assay procedures were carried out according to the application instructions provided by the manufacturer. In addition, the ELISA assay was implemented according to the kit instructions supplied by the manufacturers.

### Follow-Up

Patients were followed up consecutively at 1 month, 3 months, 6 months, 1 year, 2 years, and 3 years, for a total of 36 months, during which the recurrence of stroke and the death of patients were registered.

### Statistical Analysis

The data analysis and graph creation were completed using SPSS 26.0 (IBM Corp., Armonk, NY, USA) and GraphPad Prism 7.02 (GraphPad Software, Inc., San Diego, CA, USA), respectively. The Mann–Whitney U test or Kruskal–Wallis H rank sum test was used to compare the expression of CDC42. The change of CDC42 expression over time was analyzed using the Friedman test. Spearman's rank correlation test examined the association between the two continuous variables. Factors related to the risk of ischemic stroke were analyzed by univariate logistic regression. Receiver operating characteristic (ROC) curves were constructed to assess the correlation of the expression of CDC42 at different time points with a recurrence risk of AIS. A two-sided value of *p* < 0.05 was taken to define the statistical significance.

## Results

### Study Flow

**Figure 1:**
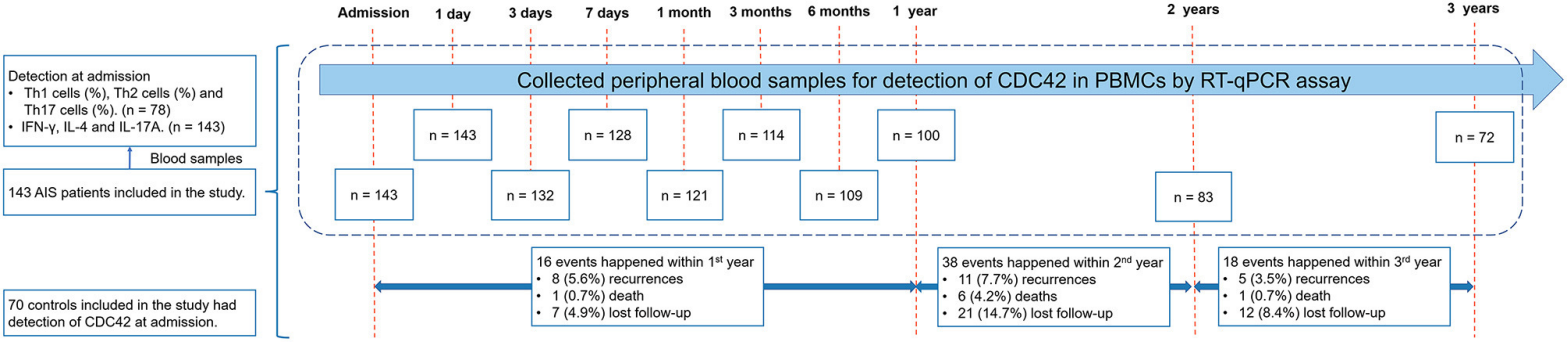
Study flow.

A total of 143 patients with AIS and 70 controls were enrolled in the study (Figure 1). For patients with AIS, CDC42, Th1, Th2, Th17 cells, IFN-γ, IL-4, and IL-17A were checked at admission. The mean time of the first sample obtained after admission was 24.9 ± 6.1 min. Then, CDC42 was additionally detected at the following time points: 1 day (*n* = 143), 3 days (*n* = 132), and 7 days (*n* = 128) after admission; 1 month (*n* = 121), 3 months (*n* = 114), 6 months (*n* = 109), 1 year (*n* = 100), 2 years (*n* = 83), and 3 years (*n* = 72) after discharge from the hospital. In addition, it should be noted that 8 (5.6%) cases recurred, 1 (0.7%) died, and 7 (4.9%) were lost a follow-up that happened within the 1st year; 11 (7.7%) recurred, 6 (4.2%) died, and 21 (14.7%) were lost a follow-up that happened within the 2nd year; and 5 (3.5%) recurred, 1 (0.7%) died, and 12 (8.4%) lost a follow-up that happened within the 3rd year. For controls, their CDC42 was observed at the time of admission.

### Characteristics of Patients With AIS

The median age of patients with AIS enrolled in this study was 64.9 ± 8.9 years, consisting of 40 (28.0%) women and 103 (72.0%) men (Table 1). Regarding the underlying diseases, 121 (84.6%), 73 (51.0%), 59 (41.3%), 31 (21.7%), and 29 (20.3%) patients suffered from hypertension, hyperlipidemia, hyperuricemia, diabetes mellitus, and chronic kidney disease, respectively. Moreover, the median NIHSS score was 7.0 (interquartile range (IQR): 4.0–11.0). According to the TOAST classification, 58 (40.6%), 34 (23.8%), 22 (15.4%), 17 (11.9%), and 12 (8.4%) patients were classified into large artery atherosclerosis (LAA), small artery occlusion (SAA), cardioembolism (CE), the stroke of undetermined etiology (SUE), and stroke of other determined etiology (SOE), respectively. In terms of the treatment, 101 (70.6%), 22 (15.4%), and 20 (14.0%) patients receive IVT, mechanical thrombectomy, and IVT coupled with mechanical thrombectomy, respectively. The detailed clinical features of patients with AIS are listed in Table 1.

**Table 1:** Characteristics of AIS patients.

**Items**	**AIS patients (*N* = 143)**
**Demographics**	
Age (years), mean ± SD	64.9 ± 8.9
Gender, *n* (%)	
Female	40 (28.0)
Male	103 (72.0)
BMI (kg/m^2^), mean ± SD	24.2 ± 2.5
History of smoke, *n* (%)	79 (55.2)
**Underlying diseases**	
Hypertension, *n* (%)	121 (84.6)
Hyperlipidemia, *n* (%)	73 (51.0)
Hyperuricemia, *n* (%)	59 (41.3)
Diabetes mellitus, *n* (%)	31 (21.7)
Chronic kidney disease, *n* (%)	29 (20.3)
**Disease features**	
NIHSS score, median (IQR)	7.0 (4.0–11.0)
TOAST classification, *n* (%)	
LAA	58 (40.6)
SAA	34 (23.8)
CE	22 (15.4)
SUE	17 (11.9)
SOE	12 (8.4)
**Laboratory examination**	
Th1 cells (%), median (IQR)	14.6 (12.9–18.4)
Th2 cells (%), median (IQR)	11.5 (9.0–14.6)
Th17 cells (%), median (IQR)	4.5 (3.9–7.1)
IFN-γ (pg/ml), median (IQR)	1.1 (0.7–2.0)
IL-4 (pg/ml), median (IQR)	15.4 (12.0–25.6)
IL-17A (pg/ml), median (IQR)	34.2 (22.6–42.8)
**Treatment**	
IVT, *n* (%)	101 (70.6)
Mechanical thrombectomy, *n* (%)	22 (15.4)
IVT combined with mechanical thrombectomy, *n* (%)	20 (14.0)

*AIS, acute ischemic stroke; SD, standard deviation; BMI, body mass index; IQR, interquartile range; NIHSS, National Institute Health of Stroke Scale; TOAST, Trial of Org 10172 in Acute Stroke Treatment; LAA, large artery atherosclerosis; SAA, small artery occlusion; CE, cardioembolism; SUE, stroke of undetermined etiology; SOE, stroke of other determined etiology; Th1 cells, T helper 1 cells; Th2 cells, T helper 2 cells; Th17 cells, T helper 17 cells; IFN-γ, interferon gamma; IL-4, interleukin-4; IL-17A, interleukin 17A; IVT, intravenous thrombolysis*.

### CDC42 Expression in Patients With AIS and Controls

**Figure 2:**
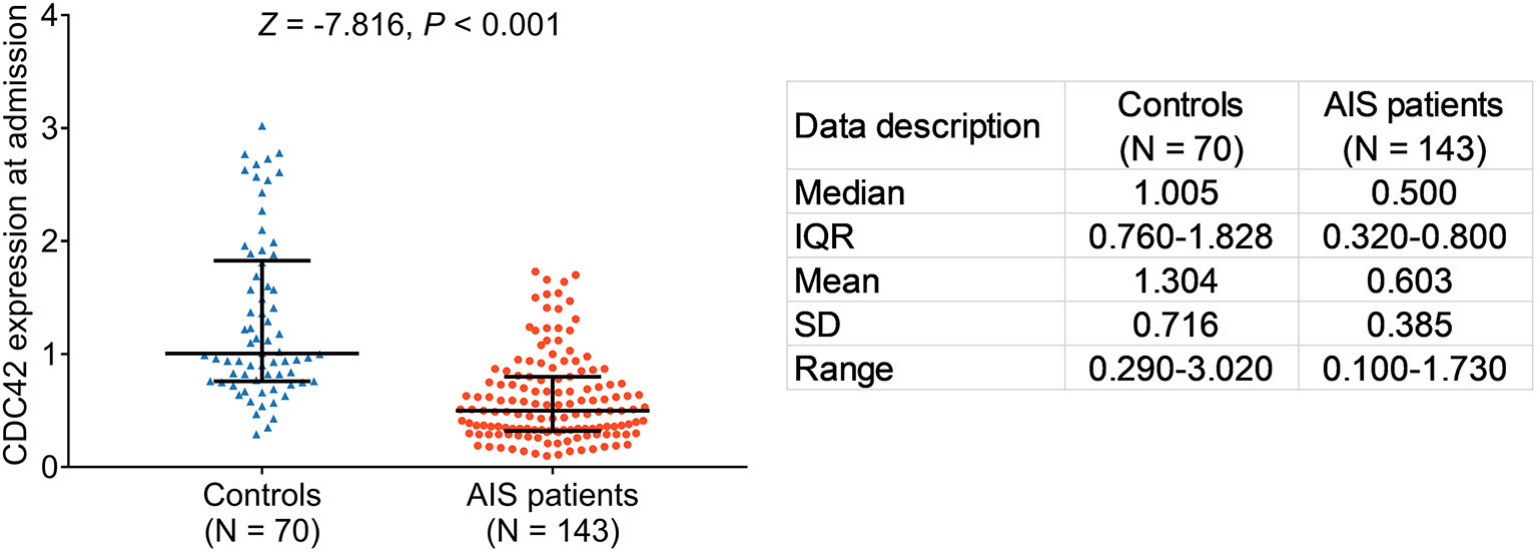
Cell division cycle 42 (CDC42) was decreased in patients with acute ischemic stroke (AIS) than in controls.

The expression of cell division cycle 42 was lower in patients with AIS than in controls (*p* < 0.001). Furthermore, the median CDC42 expression in patients with AIS and in controls was 0.500 (IQR: 0.320–0.800) and 1.005 (IQR: 0.760–1.828), respectively. In addition, the mean CDC42 expression in patients with AIS and in controls was 0.603 ± 0.385 and 1.304 ± 0.716, respectively (Figure 2). Furthermore, there was no difference in the expression of CDC42 among patients with LAA, SAA, CE, SUE, and SOE (*p* = 0.428, Supplementary Figure S1).

Additionally, the correlation of CDC42 with ischemic stroke was further varied in the logistic regression model analysis, which showed that higher CDC42 was related to the decreased risk of ischemic stroke (odds ratio (OR): 0.086, *p* < 0.001, Supplementary Table S1).

### Correlation of CDC42 With Disease Severity and Underlying Diseases in Patients With AIS

**Figure 3:**
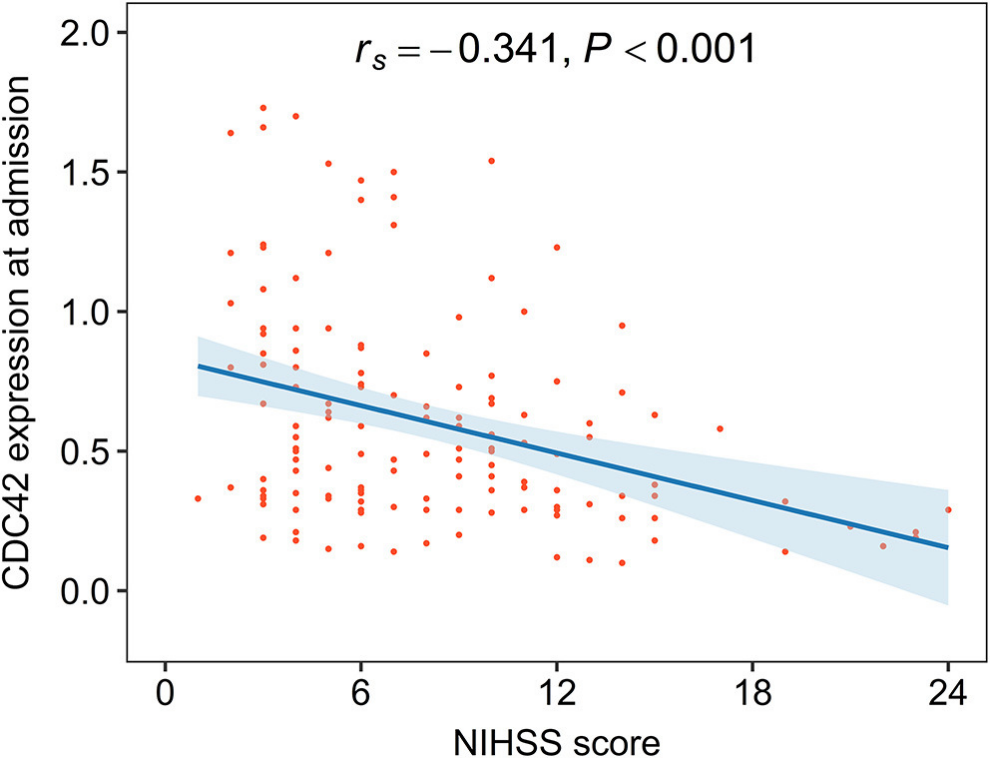
CDC42 was negatively associated with the National Institutes of Health Stroke Scale (NIHSS) score in patients with AIS.

Cell division cycle 42 was negatively correlated with the NIHSS score in patients with AIS (*r*_*s*_= −0.341, *p* < 0.001), which suggested that CDC42 might be negatively related to the overall disease severity in patients with AIS (Figure 3). To lessen statistical bias, the expression of CDC42 was compared among patients who were divided into five grades, showing that CDC42 was the highest in mild patients, followed by moderate patients, then in moderate-to-severe patients, and the lowest in severe patients (*p* < 0.001) (Supplementary Figure S2).

Moreover, CDC42 was not associated with hypertension (*p* = 0.057), hyperlipidemia (*p* = 0.115), hyperuricemia (*p* = 0.870), diabetes mellitus (*p* = 0.263), or chronic kidney disease (*p* = 0.451) in patients with AIS (Supplementary Table S2).

### Correlation of CDC42 With Th1/2/17 Cells, and Their Secreted Cytokines in Patients With AIS

**Figure 4:**
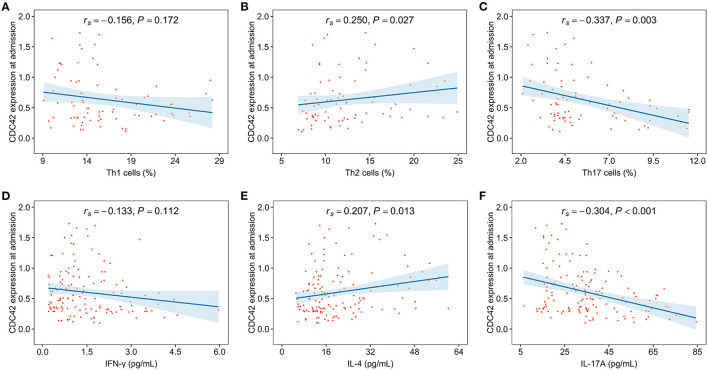
CDC42 was associated with T helper (Th)2, Th17 cells, and their secreted cytokines in patients with AIS. The correlation of CDC42 with Th1 cells **(A)**, Th2 cells **(B)**, Th17 cells **(C)**, interferon-gamma (IFN-γ) **(D)**, interleukin-4 (IL-4) **(E)**, and interleukin-17A (IL-17A) **(F)** in patients with AIS.

Cell division cycle 42 was neither related to Th1 cells (*r*_*s*_= −0.156, *p* = 0.172) nor its secreted cytokine IFN-γ (*r*_*s*_= −0.133, *p* = 0.112). However, CDC42 was positively linked with Th2 cells (*r*_*s*_= 0.250, *p* = 0.027) and its secreted cytokine IL-4 (*r*_*s*_= 0.207, *p* = 0.013) in patients with, whereas CDC42 was negatively associated with Th17 cells (*r*_*s*_= −0.337, *p* = 0.003) and its secreted cytokine IL-17A (*r*_*s*_= −0.304, *p* < 0.001) (Figures 4A–F).

### Longitudinal Changes of CDC42 Expression in Patients With AIS

**Figure 5:**
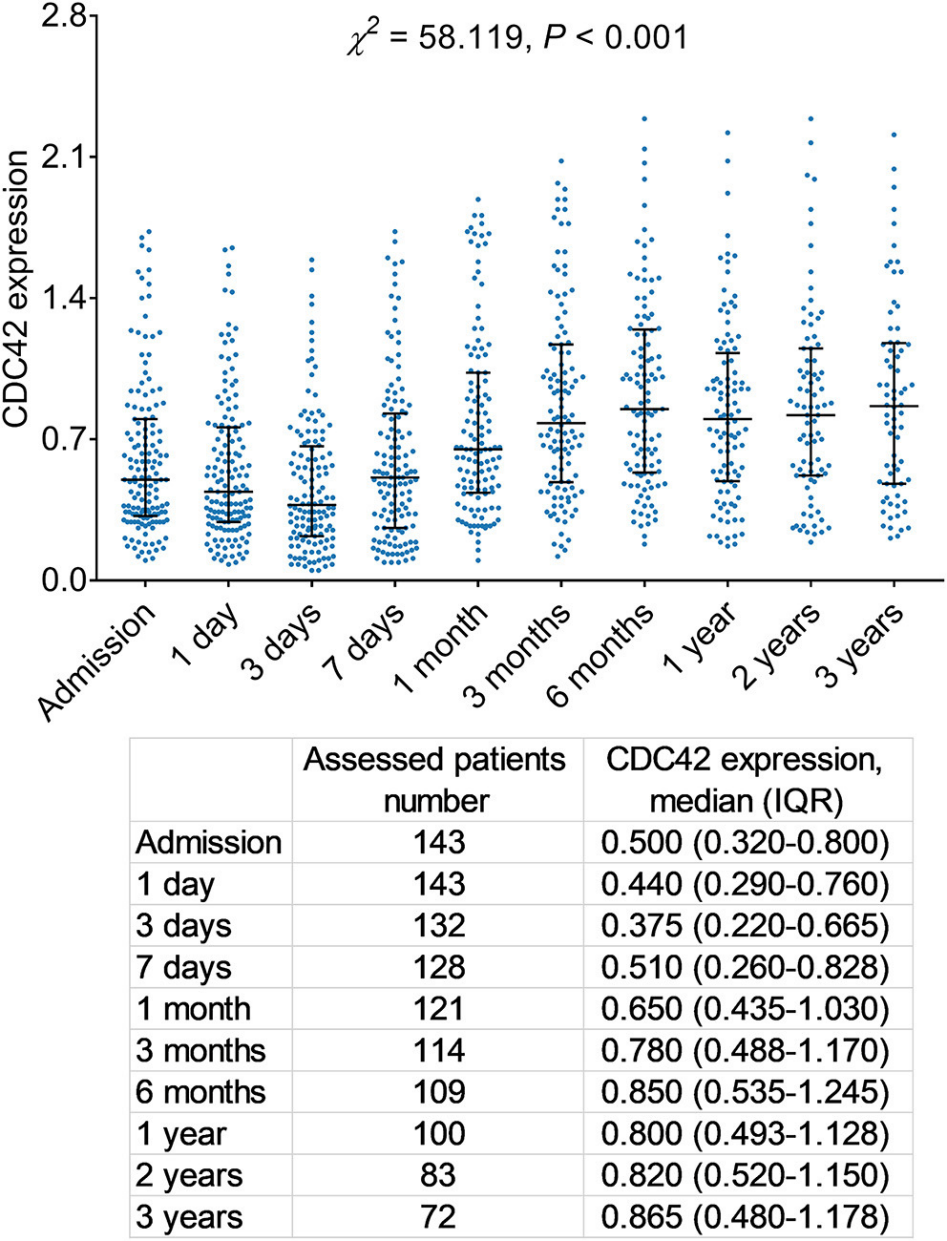
Differences of CDC42 at different time points after AIS initiation.

Overall, there are differences in CDC42 in patients with AIS at different time points (*p* < 0.001, Figure 5). Specifically, in patients with AIS, CDC42 showed a gradual decrease from admission [0.500 (IQR: 0.320–0.800)] to 3 days [0.375 (IQR: 0.220–0.665)], then it showed an increasing trend from 3 days to 3 years [0.865 (IQR: 0.480–1.178)]. Furthermore, CDC42 at 3 days was significantly reduced than that on admission (*p* < 0.001) while CDC42 at 3 years was significantly increased than that at 3 days (*p* < 0.001) (Supplementary Figure S3).

### Differences of CDC42 Between AIS Patients With mRS Score ≤ 2 and mRS Score > 2 at Each Time Point

Cell division cycle 42 CDC42 at admission (*p* = 0.013), 1 day (*p* = 0.009), 3 days (*p* = 0.001), 7 days (*p* = 0.002), 1 month (*p* = 0.004), 3 months (*p* < 0.001), 6 months (*p* = 0.005), 1 year (*p* = 0.007) was decreased in the mRS score > 2 patients than that in the mRS score ≤ 2 patients while CDC42 at 2 years (*p* = 0.099) and 3 years (*p* = 0.270) showed no difference between the patients in those two groups (Supplementary Table S3).

### Correlation of CDC42 Expression With Recurrence and Death of Patients With AIS

**Figure 6:**
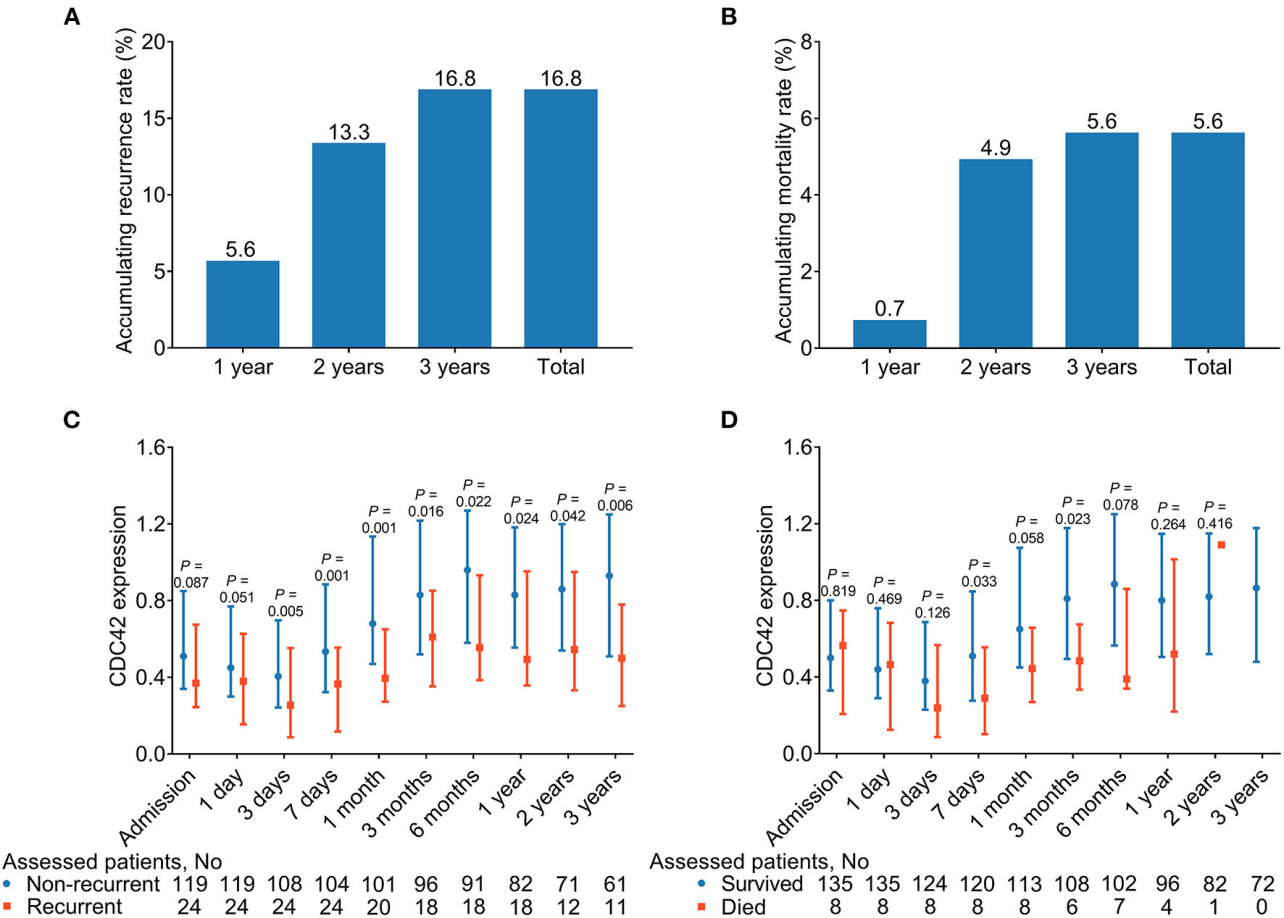
CDC42 was decreased in recurrent patients (vs. non-recurrent patients) and deceased patients (vs. survived patients) at some time points. Accumulating 1-, 2-, and 3-year, total recurrent **(A)** and mortality **(B)** rates in patients with AIS. Comparison of CDC42 at different time points in recurrent patients (vs. non-recurrent patients) **(C)** and deceased patients (vs. survived patients) **(D)**.

One-, two-, and three-year cumulative recurrence rates for patients with AIS in this study were 5.6%, 13.3%, and 16.8%, respectively. Consequently, the aggregate cumulative recurrence rate was 16.8% (Figure 6A). One-, two-, and 3three-year cumulative mortality rates for patients with AIS in this study were 0.7%, 4.9%, and 5.6%, respectively. Hence, the total accumulated mortality rate was 5.6% (Figure 6B).

Additionally, CDC42 was reduced in recurrent patients at 3 days (*p* = 0.005), 7 days (*p* = 0.001), 1 month (*p* = 0.001), 3 months (*p* = 0.016), 6 months (*p* = 0.022), 1 year (*p* = 0.024), 2 years (*p* = 0.042), and 3 years (*p* = 0.006) compared with non-recurrent patients (Figure 6C). In addition, CDC42 at 7 days (*p* = 0.033) and 3 months (*p* = 0.023) was declined in deceased patients than survived patients, while it was of no difference at other time points between those two groups (all *p* > 0.050) (Figure 6D). In detail, it was found that although CDC42 in recurrent and non-recurrent patients with AIS was declined within 3 days, then exhibited a climbing trend from 3 days to 3 years, the difference in CDC42 between these two groups was gradually enlarged over time. Meanwhile, CDC42 displayed a similar trend between survived and deceased patients with AIS to some extent.

In addition, CDC42 at some time points was decreased in 1-, 2-, and 3-year recurrent patients (vs. non-recurrent patients) (all *p* < 0.050) (Supplementary Table S4). Additionally, ROC curves showed that CDC42 at different time points, to some extent, was correlated with the recurrence risk of patients with AIS (Supplementary Figures S4A–J). Furthermore, CDC42 in a small part of the time points was declined in 1-, 2-, and 3-year deceased patients (vs. survived patients) (all *p* < 0.050) (Supplementary Table S5).

## Discussion

Cell division cycle 42, a small Rho GTPase, is related to neuronal morphology, T-cell differentiation, vascular permeability, etc. in several *in vivo* and *in vitro* studies; however, its clinical application in cerebrovascular diseases is still rare. Meanwhile, there is no clinical study focusing on the correlation between CDC42 and disease severity in patients with AIS (28–30). In this study, CDC42 was negatively related to the NIHSS score of patients with AIS, which reflected a negative correlation between CDC42 and disease severity in patients with AIS. Possible explanations might be as follows: (1) CDC42 promoted the regeneration of vascular endothelial cells (ECs) whose injury was associated with elevated vascular hyperpermeability and increased NIHSS score in patients with AIS (31, 32). (2) CDC42 was considered to promote the activated microglia returning to ramify the status, which would be helpful to cope with the injury induced by neuroinflammation. Meanwhile, CDC42 attenuated neuroinflammation *via* decreasing peripheral immune cell infiltration (33, 34). As to the systemic inflammation, CDC42 prevented the sustained upregulation of pro-inflammatory genes by reducing macrophage recruitment, and inhibiting excessive inflammation would facilitate vascular healing (35–37). (3) CDC42 might reduce the accumulation of low-density lipoproteins *via* enhancing foam cell formation, resulting in a more severe situation in patients with AIS (8, 38). Thus, CDC42 was negatively related to disease severity in patients with AIS.

Cell division cycle 42 is a key regulator of immune cell homeostasis and T-cell differentiation, according to some *in vivo* and *in vitro* studies (39, 40). For instance, one study has concluded that the native T cells lacking CDC42 accelerated differentiation into Th1 cells and CD8^+^ effector cells (39). In addition, clinical research suggests that CDC42 is positively correlated with Th2 cells but negatively correlates with Th17 cells in patients with coronary heart disease (20). However, there are no relevant clinical studies showing the involvement of CDC42 in Th1, Th2, Th17 cells and their secreted cytokines in patients with AIS. The current study revealed that CDC42 was positively linked with Th2 cells and their secreted cytokine IL-4 but negatively associated with Th17 cells and its secreted cytokine IL-17A in patients with AIS; however, CDC42 was neither related to Th1 cells nor its secreted cytokine IFN-γ. Probable reasons might be that: (1) CDC42 selectively promoted the differentiation of T cells into Th2 cells *via* 4–1BB and nucleic acid metabolism signaling pathways but suppressed T-cell differentiation into Th17 cells by diminishing glycolysis (40, 41). Consequently, CDC42 was positively related to Th2 cells and IL-4 (Th2 secreted cytokine) but negatively linked with Th17 cells and IL-17A (Th17 secreted cytokine) in patients with AIS. (2) CDC42 mainly enhanced the differentiation of T cells into Th2 cells, and the proportion of Th1 cells was indirectly affected; thus, CDC42 was positively correlated with Th2 cells and IL-4 but not linked with Th1 cells or IFN-γ in patients with AIS. In addition, it could be noticed that IFN-γ, IL-4, and IL-17A of all 143 patients with AIS were detected on admission, while only 78 Th1, Th2, and Th17 cells were detected simultaneously. The reason was that inflammation cytokines could be retained in samples for further measurement. Hence, inflammation cytokines were identified in all patients; however, the detection of Th cells in freshly isolated PBMC should be performed immediately, which some patients could not accomplish. Therefore, Th cells were detected in parts of patients with AIS.

Although various biomarkers for the management of patients with AIS (such as endostatin, sphingosine 1-phosphate, and fatty acid-binding protein 4) have been noticed in some studies, most of them are detected at a single point and multipoint detection study in the long term is rare (42–44). This study detected the expression of CDC42 expression at admission, 1 day, 3 days, 7 days, 1 month, 3 months, 6 months, 1 year, 2 years, and 3 years in patients with AIS and revealed that CDC42 gradually decreased from the day of admission to 3 days, then it showed an increasing trend from 3 days to 3 years in patients with AIS; additionally, CDC42 in recurrent, non-recurrent, survived, and deceased patients with AIS presented the similar longitudinal-change trend. A probable reason might be that stroke injury had a hysteresis effect, which meant that the stroke injury of patients with AIS is initially exacerbated, subsequently facilitated by the elimination of arterial occlusion and the restoration of blood flow to the brain (45). Considering the role of CDC42 on thrombosis and as a protective factor in AIS; therefore, CDC42 slightly decreased within 3 days and then exhibited a climbing trend from 3 days to 3 years in patients with AIS (23). Moreover, CDC42 expression data of several patients were omitted at some time points, which could explain that this was not an intervention study. Patients were monitored regularly according to the needs of their disease condition; therefore, it could not be ensured that patients could revisit on time at every time point.

In addition to the longitudinal changes, we also noted that CDC42 was lower in recurrent than in non-recurrent patients with AIS at most time points. This difference gradually widened over time, but it was quite insignificant in survived and deceased patients. The possible explanations might be as follows: (1) CDC42 contributed to blood vessel regeneration and functional recovery, whereas recurrent patients with AIS were frequently accompanied by worse vascular conditions due to thrombosis (28). Hence, CDC42 was lower in recurrent patients than in non-recurrent patients. (2) CDC42 improved neuronal function by regulating actin polymerization state and neuron cytoskeleton. Meanwhile, recurrent patients with AIS had more neuronal cell damage (46, 47). Therefore, CDC42 was reduced in recurrent patients than in non-recurrent patients. (3) CDC42 attenuated the inflammation level, which was preeminent in recurrent patients with AIS (10, 48–50). Thus, CDC42 was negatively linked with the recurrence rate in patients with AIS. (4) The relatively small number of fatal events resulted in low statistical power. As a result, the difference between CDC42 in survived and deceased patients was relatively insignificant.

There were certain limitations to this study. First, the number of patients in the current study was relatively small (*n* = 143); therefore, studies with a larger sample size to validate the findings were necessary. Second, some patients lost a follow-up over time, which might affect the results of this study. Third, this study only evaluated the clinical role of CDC42 in patients with AIS but lacked the exploration of other stroke types (such as hemorrhagic stroke).

In conclusion, CDC42 correlates with disease severity, Th2 cells, Th17 cells, and their secreted cytokines, which also serve as a biomarker to constantly monitor disease progression and recurrence risk of patients with AIS.

## Data Availability Statement

The original contributions presented in the study are included in the article/Supplementary Material, further inquiries can be directed to the corresponding author/s.

## Ethics Statement

The studies involving human participants were reviewed and approved by ShanXi Province People's Hospital of Shanxi Medical University. The patients/participants provided their written informed consent to participate in this study.

## Author Contributions

JY conceived and designed this study. XC, XZ, and KM collected and analyzed the data and wrote this manuscript. XC and JY revised this manuscript. All authors read and approved the submitted version.

## Conflict of Interest

The authors declare that the research was conducted in the absence of any commercial or financial relationships that could be construed as a potential conflict of interest.

## Publisher's Note

All claims expressed in this article are solely those of the authors and do not necessarily represent those of their affiliated organizations, or those of the publisher, the editors and the reviewers. Any product that may be evaluated in this article, or claim that may be made by its manufacturer, is not guaranteed or endorsed by the publisher.

## Supplementary Material

Supplementary Figure S1 Acute ischemic stroke (AIS) patients with different subtypes showed no difference in Cell division cycle 42 (CDC42).

Supplementary Figure S2 Comparison of CDC42 in AIS patients with different disease severity.

Supplementary Figure S3 Comparison of CDC42 to admission vs. 3 days, and 3 days vs. 3 years in patients with AIS.

Supplementary Figure S4 CDC42 was correlated with recurrence risk in patients with AIS. The correlation of CDC42 at admission **(A)**, 1 day **(B)**, 3 days **(C)**, 7 days **(D)**, 1 month **(E)**, 3 months **(F)**, 6 months **(G)**, 1 year **(H)**, 2 years **(I)**, and 3 years **(J)** with recurrence risk in patients with AIS.

Supplementary Table S1 Factors related to risk of ischemic stroke by univariate logistic regression model analysis.

Supplementary Table S2 Correlation of CDC42 at admission with underlying diseases in AIS patients.

Supplementary Table S3 Differences of CDC42 at each time point between AIS patients with mRS score ≤2 and mRS score >2.

Supplementary Table S4 Difference of CDC42 expression at each follow-up point between recurrent patients and non-recurrent patients.

Supplementary Table S5 Difference of CDC42 expression at each follow-up point between died patients and survived patients.
