# Supplementary figures and images for: Longitudinal Variations of CDC42 in Patients With Acute Ischemic Stroke During 3-Year Period: Correlation With CD4+ T Cells, Disease Severity, and Prognosis
Source: Front Neurol. 2022 Apr 25;13:848933. doi: 10.3389/fneur.2022.848933 (PMC9081787; doi:10.3389/fneur.2022.848933)

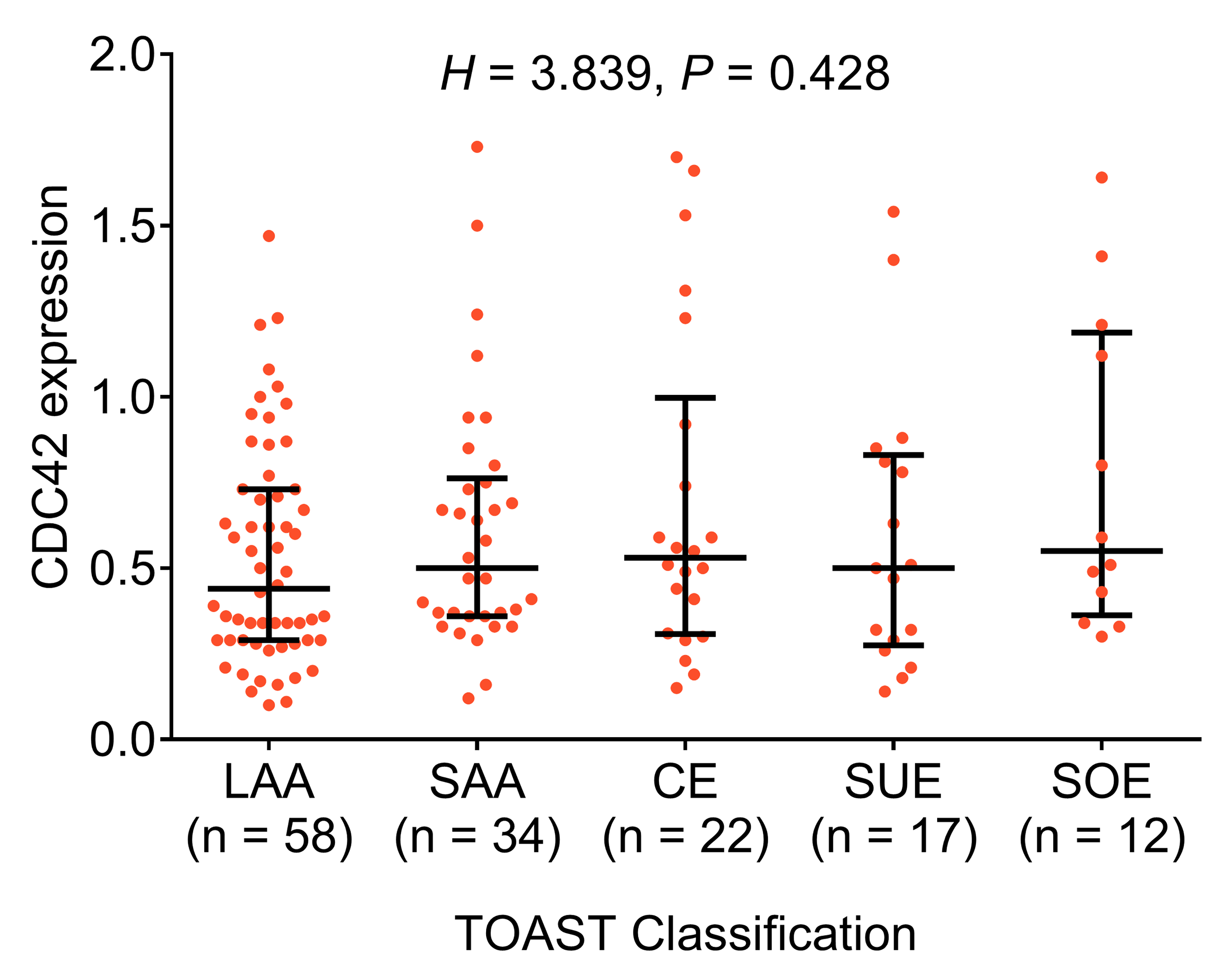

Supplement: Supplementary Figure S1 — Acute ischemic stroke (AIS) patients with different subtypes showed no difference in Cell division cycle 42 (CDC42). [file Image_1.TIF]

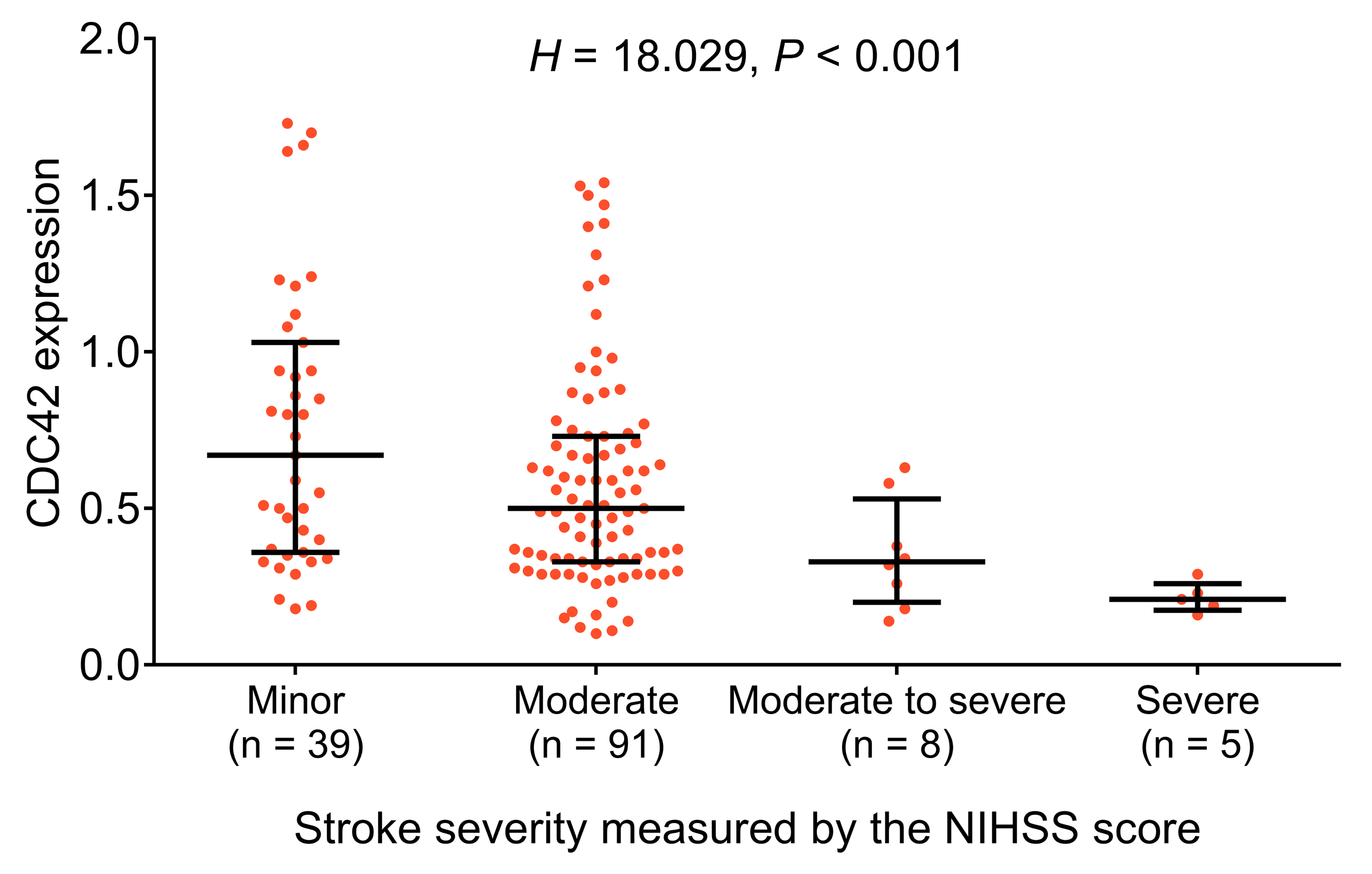

Supplement: Supplementary Figure S2 — Comparison of CDC42 in AIS patients with different disease severity. [file Image_2.TIF]

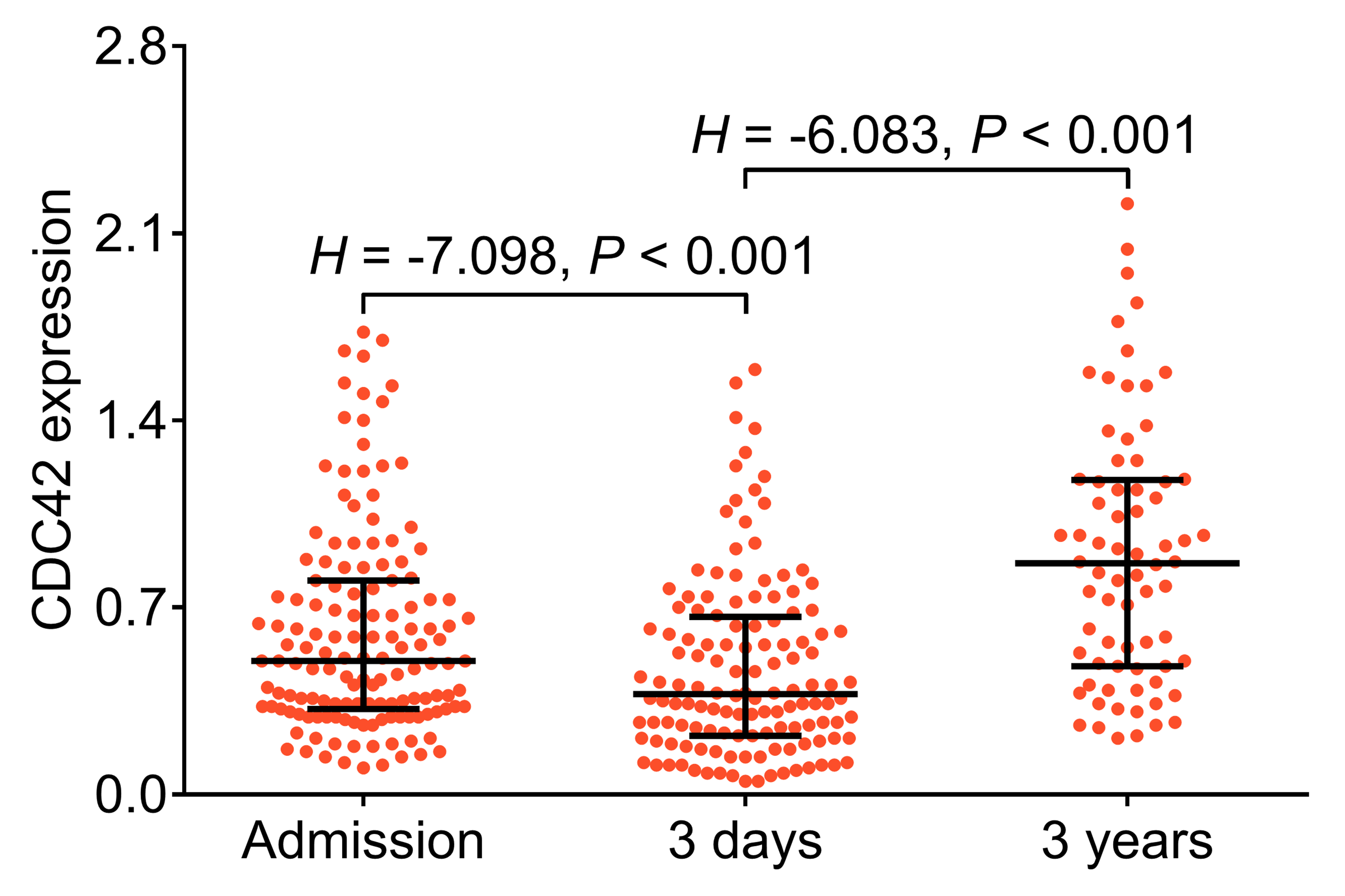

Supplement: Supplementary Figure S3 — Comparison of CDC42 to admission vs. 3 days, and 3 days vs. 3 years in patients with AIS. [file Image_3.TIF]

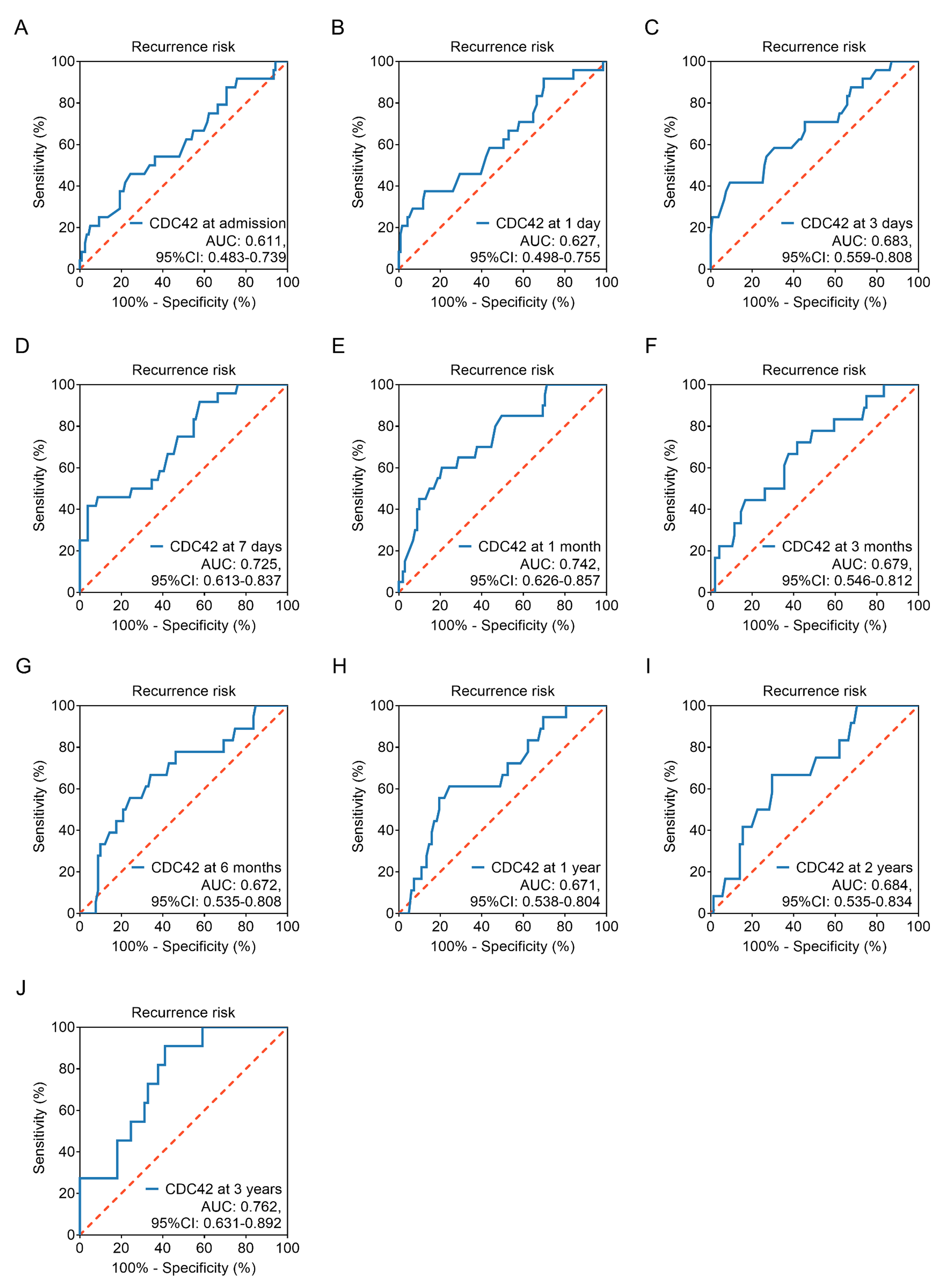

Supplement: Supplementary Figure S4 — CDC42 was correlated with recurrence risk in patients with AIS. The correlation of CDC42 at admission (A), 1 day (B), 3 days (C), 7 days (D), 1 month (E), 3 months (F), 6 months (G), 1 year (H), 2 years (I), and 3 years (J) with recurrence risk in patients with AIS. [file Image_4.TIF]
