# Supplementary material for: Longitudinal Variations of CDC42 in Patients With Acute Ischemic Stroke During 3-Year Period: Correlation With CD4+ T Cells, Disease Severity, and Prognosis
Source: Front Neurol. 2022 Apr 25;13:848933. doi: 10.3389/fneur.2022.848933 (PMC9081787; doi:10.3389/fneur.2022.848933)
Supplement: Supplementary Table S1 — Factors related to risk of ischemic stroke by univariate logistic regression model analysis. [file Table_1.docx]

**Supplementary Table 1.** Factors related to risk of ischemic stroke by univariate logistic regression model analysis.

| Items | *P* value | OR | 95%CI | |
| --- | --- | --- | --- | --- |
|  |  |  | Lower | Upper |
| Higher CDC42 | **<0.001** | 0.086 | 0.040 | 0.186 |
| Higher age | 0.732 | 1.006 | 0.973 | 1.040 |
| Gender (Male vs. Female) | 0.175 | 1.522 | 0.829 | 2.792 |
| Higher BMI | **0.018** | 1.141 | 1.022 | 1.273 |
| History of smoke (Yes vs. No) | 0.742 | 1.101 | 0.620 | 1.954 |
| Hypertension (Yes vs. No) | 0.117 | 1.764 | 0.867 | 3.590 |
| Hyperlipidemia (Yes vs. No) | 0.403 | 0.782 | 0.440 | 1.391 |
| Hyperuricemia (Yes vs. No) | **0.009** | 2.371 | 1.238 | 4.539 |
| Diabetes mellitus (Yes vs. No) | 0.440 | 1.338 | 0.640 | 2.798 |
| Chronic kidney disease (Yes vs. No) | 0.187 | 1.724 | 0.767 | 3.875 |

OR, odds ratio; CI, confidence interval; CDC42, cell division cycle 42; BMI, body mass index.
