# Supplementary material for: Longitudinal Variations of CDC42 in Patients With Acute Ischemic Stroke During 3-Year Period: Correlation With CD4+ T Cells, Disease Severity, and Prognosis
Source: Front Neurol. 2022 Apr 25;13:848933. doi: 10.3389/fneur.2022.848933 (PMC9081787; doi:10.3389/fneur.2022.848933)
Supplement: Supplementary Table S2 — Correlation of CDC42 at admission with underlying diseases in AIS patients. [file Table_2.docx]

**Supplementary Table 2.** Correlation of CDC42 at admission with underlying diseases in AIS patients.

| Items | CDC42 expression, median (IQR) | Statistic (*Z*) | *P* value |
| --- | --- | --- | --- |
| Hypertension |  | -1.903 | 0.057 |
| No | 0.590 (0.423-1.008) |  |  |
| Yes | 0.470 (0.305-0.760) |  |  |
| Hyperlipidemia |  | -1.575 | 0.115 |
| No | 0.520 (0.340-0.943) |  |  |
| Yes | 0.500 (0.290-0.720) |  |  |
| Hyperuricemia |  | -0.164 | 0.870 |
| No | 0.495 (0.333-0.730) |  |  |
| Yes | 0.510 (0.290-0.870) |  |  |
| Diabetes mellitus |  | -1.120 | 0.263 |
| No | 0.550 (0.333-0.808) |  |  |
| Yes | 0.450 (0.300-0.730) |  |  |
| Chronic kidney disease |  | -0.753 | 0.451 |
| No | 0.510 (0.330-0.800) |  |  |
| Yes | 0.370 (0.290-0.810) |  |  |

CDC42, cell division cycle 42; AIS, acute ischemic stroke; IQR, interquartile range.
