# Supplementary material for: Longitudinal Variations of CDC42 in Patients With Acute Ischemic Stroke During 3-Year Period: Correlation With CD4+ T Cells, Disease Severity, and Prognosis
Source: Front Neurol. 2022 Apr 25;13:848933. doi: 10.3389/fneur.2022.848933 (PMC9081787; doi:10.3389/fneur.2022.848933)
Supplement: Supplementary Table S3 — Differences of CDC42 at each time point between AIS patients with mRS score ≤2 and mRS score >2. [file Table_3.docx]

**Supplementary Table 3.** Differences of CDC42 at each time point between AIS patients with mRS score ≤2 and mRS score > 2

| Time points | mRS score ≤2 | mRS score >2 | Statistic (*Z*) | *P* value |
| --- | --- | --- | --- | --- |
|  | CDC42 expression, median (IQR) | |  |  |
| Admission | 0.505 (0.340-0.868) | 0.390 (0.210-0.630) | -2.476 | 0.013 |
| 1 day | 0.480 (0.300-0.780) | 0.360 (0.200-0.540) | -2.600 | 0.009 |
| 3 days | 0.415 (0.243-0.715) | 0.255 (0.110-0.375) | -3.192 | 0.001 |
| 7 days | 0.540 (0.315-0.890) | 0.395 (0.148-0.518) | -3.160 | 0.002 |
| 1 month | 0.700 (0.460-1.150) | 0.515 (0.300-0.665) | -2.865 | 0.004 |
| 3 months | 0.900 (0.620-1.280) | 0.450 (0.285-0.680) | -3.933 | <0.001 |
| 6 months | 0.985 (0.595-1.293) | 0.570 (0.370-0.875) | -2.832 | 0.005 |
| 1 year | 0.840 (0.565-1.185) | 0.490 (0.370-0.950) | -2.693 | 0.007 |
| 2 years | 0.860 (0.540-1.200) | 0.555 (0.333-0.990) | -1.651 | 0.099 |
| 3 years | 0.870 (0.480-1.233) | 0.745 (0.380-0.963) | -1.102 | 0.270 |

CDC42, cell division cycle 42; mRS, modified Rankin Scale; AIS, acute ischemic stroke; IQR, interquartile range.
