# Supplementary material for: Longitudinal Variations of CDC42 in Patients With Acute Ischemic Stroke During 3-Year Period: Correlation With CD4+ T Cells, Disease Severity, and Prognosis
Source: Front Neurol. 2022 Apr 25;13:848933. doi: 10.3389/fneur.2022.848933 (PMC9081787; doi:10.3389/fneur.2022.848933)
Supplement: Supplementary Table S4 — Difference of CDC42 expression at each follow-up point between recurrent patients and non-recurrent patients. [file Table_4.docx]

**Supplementary Table 4.** Difference of CDC42 expression at each follow-up point between recurrent patients and non-recurrent patients.

| Follow-up time | 1-year non-recurrent patients | | 1-year recurrent patients | | *P* value | 2-year non-recurrent patients | | 2-year recurrent patients | | *P* value | 3-year non-recurrent patients | | 3-year recurrent patients | | *P* value |
| --- | --- | --- | --- | --- | --- | --- | --- | --- | --- | --- | --- | --- | --- | --- | --- |
|  | Assessed patients, No. | CDC42 expression, median (IQR) | Assessed patients, No. | CDC42 expression, median (IQR) |  | Assessed patients, No. | CDC42 expression, median (IQR) | Assessed patients, No. | CDC42 expression, median (IQR) |  | Assessed patients, No. | CDC42 expression, median (IQR) | Assessed patients, No. | CDC42 expression, median (IQR) |  |
| Admission | 135 | 0.510 (0.330-0.810) | 8 | 0.305 (0.165-0.643) | 0.054 | 124 | 0.505 (0.330-0.808) | 19 | 0.380 (0.180-0.690) | 0.147 | 119 | 0.510 (0.340-0.850) | 24 | 0.370 (0.245-0.675) | 0.087 |
| 1 day | 135 | 0.450 (0.300-0.770) | 8 | 0.260 (0.125-0.470) | 0.029 | 124 | 0.450 (0.300-0.770) | 19 | 0.380 (0.120-0.620) | 0.075 | 119 | 0.450 (0.300-0.770) | 24 | 0.380 (0.155-0.628) | 0.051 |
| 3 days | 124 | 0.385 (0.230-0.688) | 8 | 0.180 (0.080-0.338) | 0.012 | 113 | 0.400 (0.235-0.690) | 19 | 0.260 (0.080-0.530) | 0.008 | 108 | 0.405 (0.243-0.698) | 24 | 0.255 (0.088-0.553) | 0.005 |
| 7 days | 120 | 0.520 (0.278-0.848) | 8 | 0.235 (0.140-0.503) | 0.023 | 109 | 0.530 (0.300-0.870) | 19 | 0.410 (0.110-0.570) | 0.007 | 104 | 0.535 (0.323-0.885) | 24 | 0.365 (0.118-0.555) | 0.001 |
| 1 month | 115 | 0.650 (0.450-1.040) | 6 | 0.440 (0.293-0.728) | 0.123 | 105 | 0.660 (0.460-1.120) | 16 | 0.440 (0.273-0.650) | 0.007 | 101 | 0.680 (0.470-1.135) | 20 | 0.395 (0.273-0.650) | 0.001 |
| 3 months | 111 | 0.790 (0.490-1.170) | 3 | 0.440 (0.390-0.000) | 0.415 | 100 | 0.805 (0.520-1.178) | 14 | 0.495 (0.275-1.028) | 0.041 | 96 | 0.830 (0.520-1.218) | 18 | 0.610 (0.353-0.853) | 0.016 |
| 6 months | 104 | 0.870 (0.543-1.248) | 5 | 0.570 (0.355-1.065) | 0.205 | 96 | 0.885 (0.555-1.248) | 13 | 0.650 (0.370-1.200) | 0.165 | 91 | 0.960 (0.580-1.270) | 18 | 0.555 (0.385-0.933) | 0.022 |
| 1 year | 95 | 0.820 (0.530-1.150) | 5 | 0.440 (0.315-0.730) | 0.074 | 86 | 0.820 (0.540-1.158) | 14 | 0.465 (0.358-0.970) | 0.050 | 82 | 0.830 (0.555-1.183) | 18 | 0.495 (0.358-0.953) | 0.024 |
| 2 years | 80 | 0.840 (0.523-1.150) | 3 | 0.520 (0.340-0.000) | 0.428 | 75 | 0.860 (0.530-1.170) | 8 | 0.545 (0.358-0.950) | 0.174 | 71 | 0.860 (0.540-1.200) | 12 | 0.545 (0.333-0.950) | 0.042 |
| 3 years | 68 | 0.870 (0.480-1.180) | 4 | 0.745 (0.365-0.975) | 0.376 | 63 | 0.920 (0.480-1.250) | 9 | 0.590 (0.415-0.805) | 0.072 | 61 | 0.930 (0.510-1.250) | 11 | 0.500 (0.250-0.780) | 0.006 |

CDC42, cell division cycle 42; IQR, interquartile range.
