# Supplementary material for: Longitudinal Variations of CDC42 in Patients With Acute Ischemic Stroke During 3-Year Period: Correlation With CD4+ T Cells, Disease Severity, and Prognosis
Source: Front Neurol. 2022 Apr 25;13:848933. doi: 10.3389/fneur.2022.848933 (PMC9081787; doi:10.3389/fneur.2022.848933)
Supplement: Supplementary Table S5 — Difference of CDC42 expression at each follow-up point between died patients and survived patients. [file Table_5.docx]

**Supplementary Table 5.** Difference of CDC42 expression at each follow-up point between died patients and survived patients.

| Follow-up time | 1-year survived patients | | 1-year died patients | | *P* value | 2-year survived patients | | 2-year died patients | | *P* value | 3-year survived patients | | 3-year died patients | | *P* value |
| --- | --- | --- | --- | --- | --- | --- | --- | --- | --- | --- | --- | --- | --- | --- | --- |
|  | Assessed patients, No. | CDC42 expression, median (IQR) | Assessed patients, No. | CDC42 expression, median (IQR) |  | Assessed patients, No. | CDC42 expression, median (IQR) | Assessed patients, No. | CDC42 expression, median (IQR) |  | Assessed patients, No. | CDC42 expression, median (IQR) | Assessed patients, No. | CDC42 expression, median (IQR) |  |
| Admission | 142 | 0.500 (0.328-0.800) | 1 | 0.180 (-) | 0.136 | 136 | 0.500 (0.330-0.800) | 7 | 0.510 (0.180-0.740) | 0.581 | 135 | 0.500 (0.330-0.800) | 8 | 0.565 (0.208-0.748) | 0.819 |
| 1 day | 142 | 0.445 (0.290-0.760) | 1 | 0.170 (-) | 0.160 | 136 | 0.445 (0.293-0.760) | 7 | 0.390 (0.110-0.700) | 0.314 | 135 | 0.440 (0.290-0.760) | 8 | 0.465 (0.125-0.683) | 0.469 |
| 3 days | 131 | 0.380 (0.220-0.670) | 1 | 0.110 (-) | 0.160 | 125 | 0.380 (0.230-0.685) | 7 | 0.140 (0.080-0.560) | 0.057 | 124 | 0.380 (0.230-0.688) | 8 | 0.240 (0.088-0.568) | 0.126 |
| 7 days | 127 | 0.510 (0.260-0.830) | 1 | 0.140 (-) | 0.151 | 121 | 0.510 (0.285-0.845) | 7 | 0.140 (0.100-0.570) | 0.032 | 120 | 0.510 (0.278-0.848) | 8 | 0.290 (0.103-0.555) | 0.033 |
| 1 month | 120 | 0.650 (0.443-1.030) | 1 | 0.270 (-) | 0.140 | 114 | 0.655 (0.450-1.058) | 7 | 0.300 (0.270-0.650) | 0.038 | 113 | 0.650 (0.450-1.075) | 8 | 0.445 (0.270-0.658) | 0.058 |
| 3 months | 113 | 0.790 (0.490-1.170) | 1 | 0.390 (-) | 0.218 | 109 | 0.810 (0.500-1.175) | 5 | 0.420 (0.280-0.620) | 0.022 | 108 | 0.810 (0.495-1.178) | 6 | 0.485 (0.335-0.675) | 0.023 |
| 6 months | 108 | 0.855 (0.543-1.248) | 1 | 0.370 (-) | 0.167 | 103 | 0.880 (0.570-1.250) | 6 | 0.380 (0.338-0.980) | 0.057 | 102 | 0.885 (0.565-1.250) | 7 | 0.390 (0.340-0.860) | 0.078 |
| 1 year | 100 | 0.800 (0.493-1.128) | 0 | - | - | 97 | 0.810 (0.510-1.145) | 3 | 0.220 (0.220-0.000) | 0.186 | 96 | 0.800 (0.505-1.148) | 4 | 0.520 (0.220-1.015) | 0.264 |
| 2 years | 83 | 0.820 (0.520-1.150) | 0 | - | - | 83 | 0.820 (0.520-1.150) | 0 | - | - | 82 | 0.820 (0.520-1.150) | 1 | 1.09 (-) | 0.416 |
| 3 years | 72 | 0.865 (0.480-1.178) | 0 | - | - | 72 | 0.865 (0.480-1.178) | 0 | - | - | 72 | 0.865 (0.480-1.178) | 0 | - | - |

CDC42, cell division cycle 42; IQR, interquartile range.
